# Periodontal Disease and Risk of Osteoradionecrosis in Patients with Head and Neck Cancer Undergoing Radiotherapy: A Systematic Review and Meta-Analysis

**DOI:** 10.3390/dj14080520

**Published:** 2026-08-14

**Authors:** Gabriela Guadalupe Zambrano Manzaba, Jossue Tarquino Narvaez Guerrero, Luis Chauca-Bajaña, Edwin Geovanny Socasi Dioses, Héctor Alfredo Lema Gutiérrez, Gema Nallely Mendoza Manzaba, Rito Alfonso Salazar Román, Andrea Ordoñez Balladares, Byron Velásquez Ron

**Affiliations:** 1 Faculty of Health Sciences, Universidad Católica de Santiago de Guayaquil (UCSG), Guayas 090101, Ecuador; gabriela.zambrano06@cu.ucsg.edu.ec (G.G.Z.M.);; 2 School of Dentistry, Universidad Católica de Santiago de Guayaquil (UCSG), Guayas 090101, Ecuador; 3 College of Dentistry, University of Guayaquil, Guayaquil 090101, Ecuador; 4 School of Medicine, Faculty of Medical Sciences, Catholic University of Santiago de Guayaquil, Guayaquil 090615, Ecuador; 5 College Dentistry, Universidad Bolivariana del Ecuador, Durán 092406, Ecuador; 6 Carrera de Odontología, Universidad de Las Américas (UDLA) Ecuador, Quito 170102, Ecuador; byron.velasquez@udla.edu.ec

**Keywords:** osteoradionecrosis, periodontal disease, periodontitis, head and neck cancer, radiotherapy, oral complications, dental management

## Abstract

**Background:** Osteoradionecrosis (ORN) is a severe late complication of radiotherapy for head and neck cancer, associated with impaired bone healing, infection, pain, and exposed bone. Periodontal disease may contribute to ORN by maintaining inflammatory and infectious foci within irradiated tissues with reduced vascularity and repair capacity. Objective: To evaluate whether periodontal disease or poor periodontal status is associated with increased ORN risk in patients with head and neck cancer undergoing radiotherapy. **Methods:** This systematic review and meta-analysis followed PRISMA 2020 recommendations and was structured according to the PECO framework. Searches were conducted in PubMed/MEDLINE, Embase, Scopus, Web of Science, Cochrane Library, ClinicalTrials.gov, WHO databases, and gray literature. Observational studies involving head and neck cancer patients treated with radiotherapy and reporting periodontal status in relation to ORN were included. Risk of bias was assessed using a domain-based approach. Random-effects models estimated pooled relative effect estimates and ORN prevalence. **Results:** Six studies were included in the primary association meta-analysis. Periodontal disease was significantly associated with increased ORN risk, with a pooled relative effect estimate of 4.83 (95% CI: 1.96–11.93; *p* = 0.0006; I^2^ = 50.4%). Seven studies including 1785 patients and 118 ORN events were included in the prevalence meta-analysis, showing a pooled ORN prevalence of 9% (95% CI: 6–13%; I^2^ = 78.0%). **Conclusions:** The findings suggest that periodontal disease or poor periodontal status may be associated with an increased likelihood of ORN in patients with head and neck cancer undergoing radiotherapy. Given the observational nature of the evidence and the moderate between-study heterogeneity, this association should be interpreted cautiously. Comprehensive periodontal assessment and long-term oral surveillance may be considered as components of multidisciplinary care before and after radiotherapy.

## 1. Introduction

Head and neck cancer remains a major oncological challenge because of its anatomical complexity, functional consequences, and the intensity of the treatments required for disease control. Radiotherapy, alone or combined with surgery and chemotherapy, is a cornerstone of treatment for many head and neck malignancies. However, irradiation of the oral cavity, salivary glands, teeth, periodontium, and jawbones may cause acute and long-term complications that negatively affect nutrition, speech, swallowing, oral function, and quality of life [1,2,3,4]. Among these complications, osteoradionecrosis (ORN) of the jaws is one of the most severe late adverse effects. ORN is generally characterized as a chronic, non-healing radiation-induced bone injury within a previously irradiated field, in the absence of persistent or recurrent tumor. Clinical manifestations may include exposed necrotic bone, pain, infection, fistula formation, trismus, impaired mastication, and pathological fracture [5,6].

The pathophysiology of ORN is complex and multifactorial. Radiation-induced vascular and cellular injury reduces tissue remodeling and healing capacity, while endothelial damage, oxidative stress, inflammation, fibroblast dysregulation, and progressive fibrosis contribute to a fibroatrophic environment [7,8]. Within these compromised tissues, additional insults such as dental infection, tooth extraction, trauma, poor oral hygiene, and persistent periodontal inflammation may facilitate the development or progression of ORN.

Periodontal diseases comprise a heterogeneous group of biofilm-mediated inflammatory conditions affecting the gingiva and the supporting tissues of the teeth. According to the current classification of periodontal and peri-implant diseases and conditions, gingivitis is characterized by clinically detectable gingival inflammation without periodontitis-related attachment loss, whereas periodontitis is defined as a chronic multifactorial inflammatory disease associated with dysbiotic dental biofilms and characterized by progressive destruction of the tooth-supporting apparatus. Periodontitis is clinically identified by interdental clinical attachment loss at two or more non-adjacent teeth, or by buccal or oral clinical attachment loss of ≥3 mm with pocketing > 3 mm at two or more teeth, provided that the attachment loss cannot be attributed to non-periodontal causes. The disease is further classified according to stage (I–IV), reflecting severity, extent, and treatment complexity, and grade (A–C), reflecting the estimated rate of progression and relevant risk modifiers [9,10,11,12].

Pre-radiotherapy dental assessment has therefore become an essential component of multidisciplinary head and neck cancer care. The aim is not only to identify teeth with poor prognosis or active infection, but also to reduce the future risk of severe oral sequelae. Nevertheless, clinical decision-making remains challenging. Extracting periodontally compromised teeth before radiotherapy may reduce the burden of infection, but it may also delay cancer treatment or create surgical wounds that need adequate healing before irradiation. Conversely, preserving teeth with advanced periodontal disease may maintain function but could leave chronic inflammatory foci inside a field that will later become hypovascular and less capable of healing [6,13].

Several observational studies have suggested that periodontal disease or poor periodontal status may be associated with ORN development, although the evidence remains heterogeneous. Goldwaser et al. reported that poor post-radiotherapy oral and periodontal conditions, including advanced radiographic periodontal deterioration, were associated with mandibular ORN [14]. Brennan et al. observed that oral foci were common before radiotherapy and that severe periodontal disease was frequently present among patients who developed ORN [15]. Lang et al. described poor dental and periodontal conditions in irradiated oral cancer patients, although periodontal variables were not always reported with extractable comparative estimates [16]. In a large cohort of oral cavity and oropharyngeal cancer patients treated with intensity-modulated radiotherapy, Akiyama et al. found that poor periodontal status was associated with ORN in univariate analysis, while radiation dose and alcohol use remained important factors in multivariable models [17]. Kojima et al. further highlighted the relevance of dental infection and severe marginal periodontitis in relation to ORN risk [18].

More recent studies have added nuance to this relationship. Kuo et al. reported that patients with advanced periodontal disease before intensity-modulated radiotherapy or chemoradiotherapy were more prone to develop bone healing problems, suggesting that periodontal inflammatory burden may be clinically meaningful even when strict ORN events are relatively uncommon [19,20]. Ito et al. introduced an imaging-based approach, showing that CT-defined stage III periodontitis and increased periodontal inflammatory uptake on pretreatment ^18^F-FDG PET/CT were associated with subsequent ORN in patients with oral cavity or oropharyngeal cancer [21]. These findings support the concept that periodontal disease may not be merely a coincidental comorbidity, but a potentially relevant local inflammatory and infectious factor in irradiated jawbone vulnerability.

Despite these observations, the strength and consistency of the association between periodontal disease and ORN remain uncertain. Previous studies differ in design, sample size, cancer site, radiotherapy modality, follow-up duration, periodontal assessment method, ORN definition, and adjustment for confounders such as radiation dose, dental extraction, alcohol use, smoking, tumor location, and baseline oral health. Moreover, some studies report ORN prevalence without providing comparative periodontal risk estimates, whereas others evaluate periodontal disease as part of broader oral health or dental infection categories. This variability makes it difficult for clinicians to determine how strongly periodontal disease should influence pre-radiotherapy dental planning and long-term periodontal maintenance [22].

Therefore, the present systematic review and meta-analysis was designed to evaluate whether periodontal disease or poor periodontal status is associated with an increased risk of osteoradionecrosis in patients with head and neck cancer undergoing radiotherapy. A secondary objective was to estimate the pooled prevalence of ORN among the included studies and to synthesize qualitative evidence regarding periodontal and oral health factors relevant to ORN development. By clarifying this relationship, this review aims to support more evidence-informed dental screening, periodontal management, and long-term oral surveillance in patients receiving radiotherapy for head and neck cancer.

## 2. Materials and Methods

### 2.1. Protocol and Registration

This systematic review and meta-analysis was conducted according to a predefined protocol established before the beginning of the literature search, study selection, and data extraction procedures. The methodology followed the recommendations of the Preferred Reporting Items for Systematic Reviews and Meta-Analyses (PRISMA) 2020 statement [23]. The review was designed to assess whether periodontal disease or poor periodontal status is associated with an increased risk of osteoradionecrosis among patients with head and neck cancer treated with radiotherapy.

**Figure 1:**
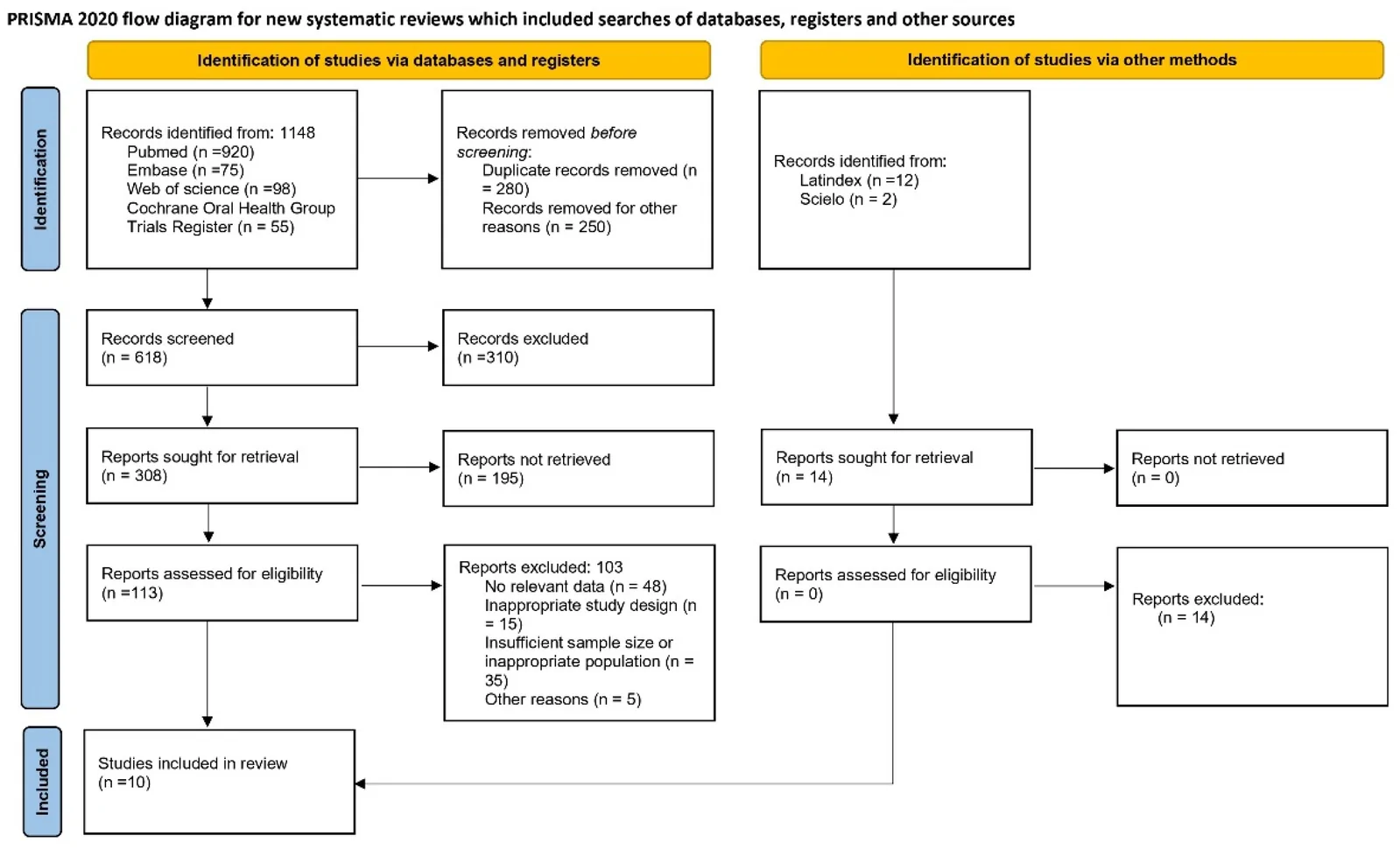
Flowchart of selected studies.

The protocol was prospectively registered in the International Prospective Register of Systematic Reviews (PROSPERO) with the registration number CRD420261440753. The PRISMA flow diagram describing the identification, screening, eligibility assessment, and inclusion of studies will be presented in Figure 1. In addition, the completed PRISMA 2020 checklist is provided as Supplementary Material (Table S1).

### 2.2. Focused Question

The focused question was formulated according to the PECO framework: Do head and neck cancer patients undergoing radiotherapy with periodontal disease or poor periodontal status have a higher risk of developing osteoradionecrosis compared with those without periodontal disease or with healthy periodontal status?

P (Population): Adult patients diagnosed with head and neck cancer and treated with radiotherapy, either alone or in combination with surgery and/or chemotherapy.

E (Exposure): Presence of periodontal disease or poor periodontal status, including gingivitis, periodontitis, periodontal attachment loss, increased probing depth, alveolar bone loss, tooth mobility, or tooth loss attributable to periodontal disease, assessed clinically, radiographically, or through validated periodontal records.

C (Comparison): Head and neck cancer patients undergoing radiotherapy without periodontal disease, with healthy periodontal status, or with mild periodontal involvement.

O (Outcome): Development of osteoradionecrosis of the jaws following radiotherapy. Secondary outcomes included the pooled prevalence of osteoradionecrosis and qualitative evidence regarding other radiotherapy-related oral complications when reported by the included studies.

### 2.3. Information Sources and Search Strategy

A comprehensive and structured literature search was conducted in accordance with the PRISMA 2020 recommendations. The search strategy was designed to identify studies evaluating the association between periodontal disease or poor periodontal status and osteoradionecrosis in patients with head and neck cancer undergoing radiotherapy. Broader radiotherapy-related oral complication terms were also included to increase search sensitivity and to identify studies reporting osteoradionecrosis within wider oral toxicity outcomes.

Electronic searches were performed in the following databases: MEDLINE via PubMed, Embase via Ovid, Scopus, Web of Science, Cochrane Library, ClinicalTrials.gov, and the regional World Health Organization databases, including LILACS, AIM, IMEMR, IMSEAR, and WPRIM. In addition, gray literature and non-indexed studies were searched through Google Scholar, ProQuest Dissertations and Theses, OpenGrey, and the Conference Proceedings Citation Index. No restrictions were applied regarding publication year. Language restrictions were applied according to the final eligibility criteria.

The search strategy combined Medical Subject Headings (MeSH), Emtree terms, and free-text keywords related to periodontal disease, head and neck cancer, radiotherapy, and osteoradionecrosis. The main search terms included: “Periodontitis”, “Periodontal Diseases”, “Gingivitis”, “Alveolar Bone Loss”, “Tooth Loss”, “Periodontal Status”, “Head and Neck Neoplasms”, “Mouth Neoplasms”, “Pharyngeal Neoplasms”, “Laryngeal Neoplasms”, “Radiotherapy”, “Radiation Therapy”, “Osteoradionecrosis”, “Jaw Osteoradionecrosis”, and “Radiation-Induced Osteonecrosis”. Additional free-text terms were incorporated to increase search sensitivity, including periodontal infection, periodontal inflammation, oral microbiota, oral pathogens, oral cancer, oropharyngeal cancer, mandibular osteoradionecrosis, osteonecrosis of the jaw, radiation-induced oral toxicity, oral mucositis, xerostomia, radiation caries, tooth loss, oral infection, and radiotherapy-related oral complications.

Boolean operators were used to combine terms as follows: periodontal disease-related terms were combined with head and neck cancer-related terms, radiotherapy-related terms, and osteoradionecrosis-related terms using AND, while synonyms and related terms were combined using OR. The search strategy was adapted for each database according to its specific indexing system and syntax.

All identified records were exported to reference management software for duplicate removal. Subsequently, the remaining records were imported into Rayyan QCRI (Qatar Computing Research Institute, Doha, Qatar) to facilitate independent screening of titles, abstracts, and full texts by reviewers.

To ensure completeness, the reference lists of all included studies and relevant reviews were manually screened. A targeted manual search was also performed in peer-reviewed journals related to periodontology, oral oncology, radiation oncology, oral medicine, maxillofacial surgery, and supportive cancer care.

### 2.4. Eligibility Criteria

The eligibility criteria were established according to the PECO framework, considering periodontal disease or poor periodontal status as the exposure and osteoradionecrosis of the jaws following radiotherapy as the primary outcome of interest.

Inclusion Criteria:

Studies were considered eligible if they met the following criteria:

1. Study design: Observational studies, including prospective cohort, retrospective cohort, case–control, nested case–control, and cross-sectional studies evaluating the association between periodontal disease or poor periodontal status and osteoradionecrosis in patients with head and neck cancer undergoing radiotherapy.
2. Population: Adult patients aged ≥18 years diagnosed with head and neck cancer and treated with radiotherapy, either alone or in combination with chemotherapy and/or surgery.
3. Exposure: Presence of periodontal disease or poor periodontal status before, during, or after radiotherapy, diagnosed through clinical periodontal parameters, radiographic findings, validated periodontal indices, imaging-based periodontal assessment, or documented periodontal history. Periodontal disease assessment could include probing depth, clinical attachment loss, bleeding on probing, plaque index, gingival index, periodontal pocket depth, alveolar bone loss, tooth mobility, severe marginal periodontitis, periodontal inflamed surface area, or tooth loss attributable to periodontal disease.
4. Comparator: Patients without periodontal disease, with healthy periodontal status, or with mild periodontal involvement. For the prevalence meta-analysis, studies reporting osteoradionecrosis events and total sample size were also considered, even when comparative periodontal effect estimates were not available.
5. Outcomes: The primary outcome was osteoradionecrosis of the jaws following radiotherapy. Secondary outcomes included the pooled prevalence of osteoradionecrosis and qualitative evidence regarding other radiotherapy-related oral complications when reported by the included studies.
6. Effect estimates: For the primary association meta-analysis, studies were eligible if they reported risk estimates such as hazard ratio, odds ratio, relative risk, prevalence ratio, or incidence rate ratio, with corresponding 95% confidence intervals, or provided sufficient raw data to calculate these measures.
7. Publication type: Full-text peer-reviewed articles.
8. Language: Articles published in English.

Exclusion Criteria:

Studies were excluded if they met any of the following criteria:

1. Clinical trials, systematic reviews, meta-analyses, narrative reviews, scoping reviews, editorials, letters to the editor, commentaries, conference abstracts without full data, case reports, or case series.
2. Animal studies, in vitro studies, laboratory-based studies, or studies involving pediatric populations.
3. Studies evaluating patients with cancers other than head and neck cancer without separate data for head and neck cancer patients.
4. Studies assessing radiotherapy-related oral outcomes without evaluating periodontal disease, periodontal status, oral health status, or dental infection as an exposure or associated factor.
5. Studies reporting general oral health, dental status, or number of teeth without sufficient periodontal attribution or without relevance to osteoradionecrosis.
6. Studies without a defined comparator group or without sufficient information to obtain or reconstruct an effect estimate for the main association analysis. However, studies reporting osteoradionecrosis events and total sample size were retained for the prevalence meta-analysis or qualitative synthesis when appropriate.
7. Studies evaluating oral complications unrelated to radiotherapy or studies in which radiotherapy exposure was not clearly reported.
8. Studies in which osteoradionecrosis was combined with other forms of jaw necrosis, such as osteochemonecrosis or medication-related osteonecrosis of the jaw, without separate extractable data for osteoradionecrosis.
9. Duplicate publications, overlapping cohorts, or secondary analyses of the same population, in which case the most complete, recent, or methodologically robust study was retained.

### 2.5. Study Selection Process and Data Extraction

Two independent reviewers (LC and AOB) screened all retrieved records and extracted data using a predesigned and standardized data extraction form. After duplicate removal, titles and abstracts were independently evaluated to identify potentially eligible studies. Full-text articles were then assessed according to the predefined inclusion and exclusion criteria. Any disagreement between the two reviewers was resolved through discussion, and when consensus could not be reached, a third reviewer (KS) was consulted. Inter-reviewer agreement during the study selection process was assessed using Cohen’s kappa (κ) coefficient.

For each eligible study, the following variables were extracted: first author and year of publication, study design, population characteristics, cancer type, sample size, radiotherapy or oncological treatment modality, follow-up duration or timing of assessment, periodontal or oral health assessment method, main outcome evaluated, number of osteoradionecrosis events, prevalence of osteoradionecrosis, and the main qualitative findings. The timing of periodontal assessment in relation to radiotherapy was also extracted and categorized as pretreatment, during radiotherapy, post-treatment follow-up, or mixed/unclear. This distinction was considered clinically relevant because baseline periodontal status represents a pre-existing exposure, whereas periodontal findings recorded during or after radiotherapy may reflect treatment-related changes, disease progression, or time-varying periodontal conditions.

Special attention was given to the method used to assess periodontal disease or poor periodontal status, including periodontal pocket depth, plaque score, alveolar bone loss, clinical attachment loss, radiographic periodontal status, panoramic radiographic scoring, computed tomography-based periodontal staging, periodontal inflamed surface area, and PET/CT-based periodontal inflammatory uptake when available.

Because the included studies used non-uniform definitions of periodontal disease and several predated the 2017 World Workshop classification, periodontal exposure was extracted according to the operational criteria reported by the original authors. No post hoc reclassification into current periodontitis stages or grades was attempted when the necessary patient-level clinical and radiographic information was unavailable. Accordingly, clinical periodontal measurements, radiographic bone-loss categories, oral infectious-focus definitions, and imaging-based inflammatory indicators were recorded as distinct methods of exposure ascertainment. In this review, the expression “periodontal disease or poor periodontal status” was therefore retained as an umbrella term encompassing the heterogeneous periodontal exposures reported in the available literature.

For the primary quantitative synthesis, studies were considered eligible if they reported an association between periodontal disease or poor periodontal status and osteoradionecrosis, either as hazard ratios, odds ratios, relative risks, or sufficient data to reconstruct an effect estimate. Studies that reported osteoradionecrosis prevalence but did not provide extractable comparative data for periodontal disease were included only in the prevalence meta-analysis or qualitative synthesis, as appropriate. Studies in which periodontal disease was evaluated as an outcome rather than an exposure, or in which osteoradionecrosis was combined with other forms of jaw necrosis without separate data, were excluded from the main association meta-analysis.

When essential data were missing, unclear, or not directly extractable from the manuscript, attempts were planned to contact the corresponding authors for clarification or additional information. The extracted characteristics of the included studies are summarized in Table 1.

Khaw et al. (2014) [27] and Morais et al. (2020) [31] were included only in the qualitative synthesis because they evaluated radiotherapy-related oral complications and preventive oral care strategies but did not provide extractable data for the periodontal disease–osteoradionecrosis association.

### 2.6. Quality Assessment and Risk of Bias Evaluation

The methodological quality and risk of bias of the included observational studies were independently assessed by two reviewers (LC and AOB) using a domain-based risk-of-bias approach adapted for non-randomized studies [34,35]. Disagreements between reviewers were resolved through discussion, and when consensus could not be reached, a third reviewer (BVR) was consulted.

The assessment considered seven methodological domains: bias due to confounding, bias in the selection of participants, bias in the classification of periodontal exposure, bias due to deviations from the intended exposure, bias due to missing data, bias in the measurement of outcomes, and bias in the selection of the reported result. Each domain was judged as presenting a low, moderate, or serious risk of bias according to the methodological characteristics and information reported in each study.

The observational or retrospective design of a study was not used as an isolated criterion to determine its risk-of-bias judgment. Therefore, retrospective studies were not automatically classified as having a moderate or serious risk of bias solely because data had been collected from existing clinical, radiographic, or imaging records. Instead, the assessment considered whether participant selection criteria were clearly defined, periodontal exposure and ORN outcomes were ascertained using documented and reproducible methods, follow-up and outcome data were sufficiently complete, missing data were adequately addressed, and the reported analyses were consistent with the study objectives.

Particular attention was given to potential confounding factors relevant to ORN risk, including radiation dose, tumor location, dental extractions, oral hygiene status, smoking, alcohol consumption, baseline dental condition, and type of oncological treatment. A low-risk judgment was assigned when no major methodological limitation was identified within the evaluated domain based on the information reported by the original study. A moderate-risk judgment was assigned when adjustment for relevant confounding factors was incomplete, periodontal exposure was assessed using non-standardized criteria, the timing of exposure assessment was unclear, or the available methodological information was insufficient to exclude some concern. Serious risk of bias was reserved for limitations considered likely to substantially compromise the validity of the reported findings.

The overall risk-of-bias judgment for each study was determined by the highest level of concern identified across the evaluated domains. Nevertheless, a low-risk judgment should not be interpreted as indicating the complete absence of residual confounding, methodological limitations, or uncertainty in the evidence. Rather, it indicates that no major source of bias was identified within most evaluated domains based on the information available in the original publication.

A graphical risk-of-bias matrix was generated to summarize the domain-level and overall judgments of the included studies. No study was excluded solely on the basis of its risk-of-bias assessment, as all studies met the predefined minimum methodological criteria for inclusion in the qualitative synthesis. Studies providing sufficient comparative data were additionally considered for the quantitative meta-analysis.

### 2.7. Statistical Analysis

All statistical analyses were performed using R software (version 4.3.1; R Foundation for Statistical Computing, Vienna, Austria) with the meta, metafor, dmetar, and ggplot2 packages. The primary quantitative synthesis evaluated the association between periodontal disease or poor periodontal status and osteoradionecrosis in patients with head and neck cancer undergoing radiotherapy.

For the association meta-analysis, the most appropriate relative effect estimates were extracted from each study, including hazard ratios, odds ratios, or risk ratios, with corresponding 95% confidence intervals when available. Because different types of relative measures were reported across the included observational studies, all estimates were treated as relative effect estimates rather than as a single effect measure. All effect estimates were transformed into the natural logarithmic scale, and standard errors were calculated from the reported confidence intervals using the formula: SE = [ln(upper CI) − ln(lower CI)]/3.92. Pooled estimates were then back-transformed and reported as pooled relative effect estimates with 95% confidence intervals.

A random-effects inverse-variance model was used because clinical and methodological heterogeneity was expected across studies, particularly regarding cancer site, radiotherapy modality, follow-up duration, periodontal assessment method, and definition of osteoradionecrosis. Between-study variance was estimated using the DerSimonian–Laird method. Statistical heterogeneity was assessed using Cochran’s Q test, τ^2^, and the I^2^ statistic. Prediction intervals were calculated to estimate the expected range of effects in future comparable clinical settings.

A secondary meta-analysis of proportions was performed to estimate the pooled prevalence of osteoradionecrosis among head and neck cancer patients undergoing radiotherapy. For this analysis, the number of osteoradionecrosis events and the total sample size were extracted from each eligible study. Pooled prevalence estimates were calculated using a random-effects model and reported as proportions with 95% confidence intervals. Heterogeneity was assessed using τ^2^, Cochran’s Q test, and I^2^.

Sensitivity analyses were conducted to evaluate the robustness of the primary findings. First, studies with extreme or influential effect estimates were excluded individually, including Katsura et al. (2008) [24], which showed the largest individual effect estimate, and Schuurhuis et al. (2018) [30], which showed the estimate closest to the null. Second, a Hartung–Knapp adjusted random-effects model was performed as a conservative sensitivity analysis because of the small number of studies included. Third, a leave-one-out analysis was conducted by sequentially excluding one study at a time and recalculating the pooled effect estimate.

Small-study effects and potential publication bias were explored visually using funnel plots. Formal statistical tests for funnel plot asymmetry, such as Egger’s test, were not emphasized because fewer than ten studies were included in the quantitative synthesis, making such tests underpowered and potentially misleading. Meta-regression and subgroup analyses were not performed because of the limited number of studies and the risk of unreliable estimates. A *p*-value < 0.05 was considered statistically significant.

## 3. Results

### 3.1. Risk of Bias Assessment

**Figure 2:**
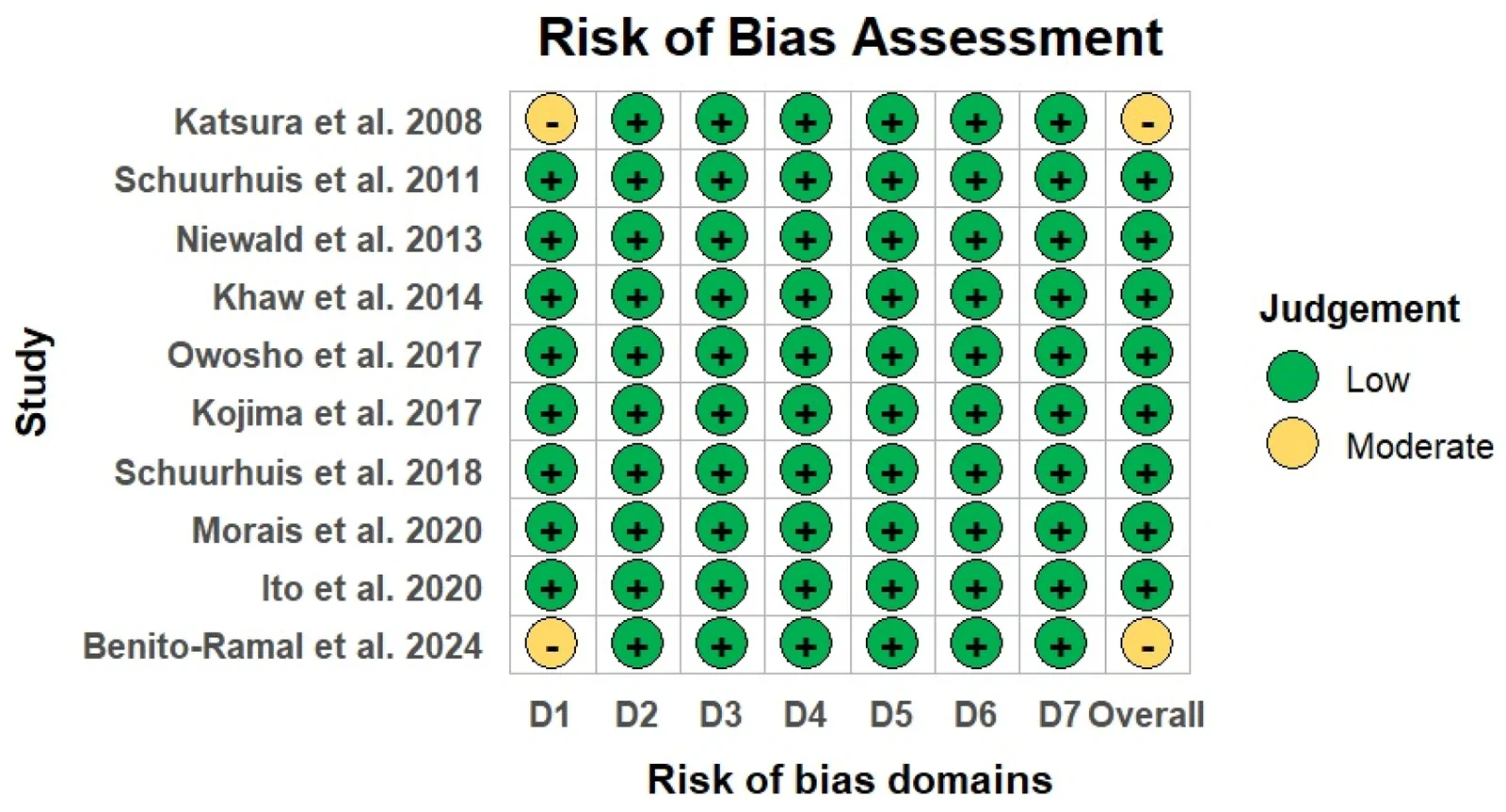
Risk of bias assessment of the included studies [24,25,26,27,28,29,30,31,32,33].

The risk of bias assessment showed that most included studies were classified as having low risk of bias across the evaluated domains. Overall, the methodological quality of the evidence was considered acceptable, with most domains judged as low risk. Moderate risk of bias was identified in selected studies, mainly in the domain related to confounding, reflecting the observational nature of the included evidence and the potential influence of clinical variables such as radiation dose, tumor location, dental extractions, oral hygiene status, smoking, alcohol consumption, and baseline dental condition. No study was classified as having serious risk of bias. These findings suggest that the included studies were generally methodologically adequate for qualitative synthesis and quantitative meta-analysis, although residual confounding should be considered when interpreting the pooled association between periodontal disease and osteoradionecrosis (Figure 2).

### 3.2. Association Between Periodontal Disease and Osteoradionecrosis

Six studies were included in the quantitative synthesis evaluating the association between periodontal disease or poor periodontal status and osteoradionecrosis in patients with head and neck cancer undergoing radiotherapy. Using a random-effects inverse-variance model, periodontal disease was significantly associated with an increased risk of osteoradionecrosis. The pooled relative effect estimate was 4.83 (95% CI: 1.96–11.93; *p* = 0.0006), indicating that periodontal disease or worse periodontal status was associated with an approximately five-fold higher likelihood of osteoradionecrosis compared with absence of periodontal disease or better periodontal status.

Individual study estimates generally favored an increased risk of osteoradionecrosis among patients with periodontal disease. The largest effect estimate was observed in Katsura et al. 2008 [24], whereas Schuurhuis et al. 2018 [30] showed a near-null association. Moderate between-study heterogeneity was observed (τ^2^ = 0.5950; χ^2^ = 10.08, df = 5; *p* = 0.073; I^2^ = 50.4%), suggesting some variability in effect estimates across studies. The prediction interval ranged from 0.48 to 48.67, indicating that although the pooled association was statistically significant, the magnitude and direction of the effect may vary across future populations and study settings.

**Figure 3:**
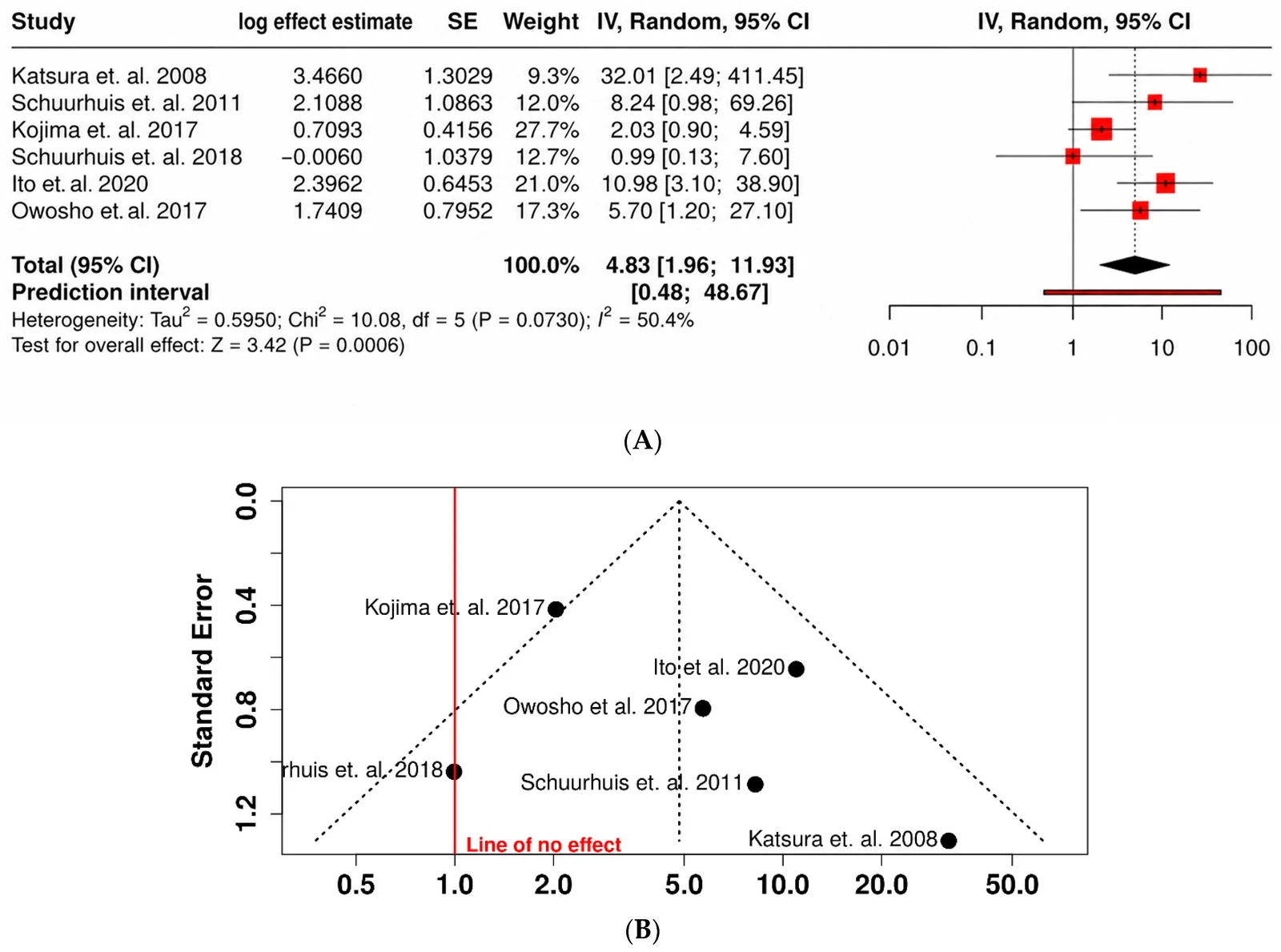
Meta-analysis of the association between periodontal disease and osteoradionecrosis in patients with head and neck cancer undergoing radiotherapy. (**A**) Forest plot showing the pooled association between periodontal disease or poor periodontal status and osteoradionecrosis. The random-effects model showed a significant positive association, with a pooled relative effect estimate of 4.83 (95% CI: 1.96–11.93; *p* = 0.0006). Moderate heterogeneity was observed across studies (I^2^ = 50.4%; τ^2^ = 0.5950). (**B**) Funnel plot assessing small-study effects. Visual inspection suggested some asymmetry, mainly driven by smaller studies with larger effect estimates and wider standard errors. Because fewer than ten studies were included, publication bias assessment should be interpreted cautiously [24,25,28,29,30,32].

Visual inspection of the funnel plot suggested some degree of asymmetry, mainly influenced by small studies with larger effect estimates and wider standard errors. However, because fewer than ten studies were included, assessment of publication bias or small-study effects should be interpreted with caution, and no definitive conclusion regarding publication bias can be established (Figure 3).

### 3.3. Pooled Prevalence of Osteoradionecrosis

A secondary meta-analysis of proportions was performed to estimate the pooled prevalence of osteoradionecrosis among patients with head and neck cancer undergoing radiotherapy. Seven studies, including 1785 patients and 118 osteoradionecrosis events, were included in this analysis. Using a random-effects model, the pooled prevalence of osteoradionecrosis was 9% (95% CI: 6–13%). Individual study estimates ranged from 4% in Owosho et al. 2017 [28] to 15% in Katsura et al. 2008 [24].

Substantial between-study heterogeneity was observed (τ^2^ = 0.0048; χ^2^ = 27.25, df = 6; *p* = 0.0001; I^2^ = 78.0%), indicating considerable variability in ORN prevalence across studies. The prediction interval ranged from 1% to 23%, suggesting that the expected prevalence of ORN may vary widely across future populations and clinical settings.

**Figure 4:**
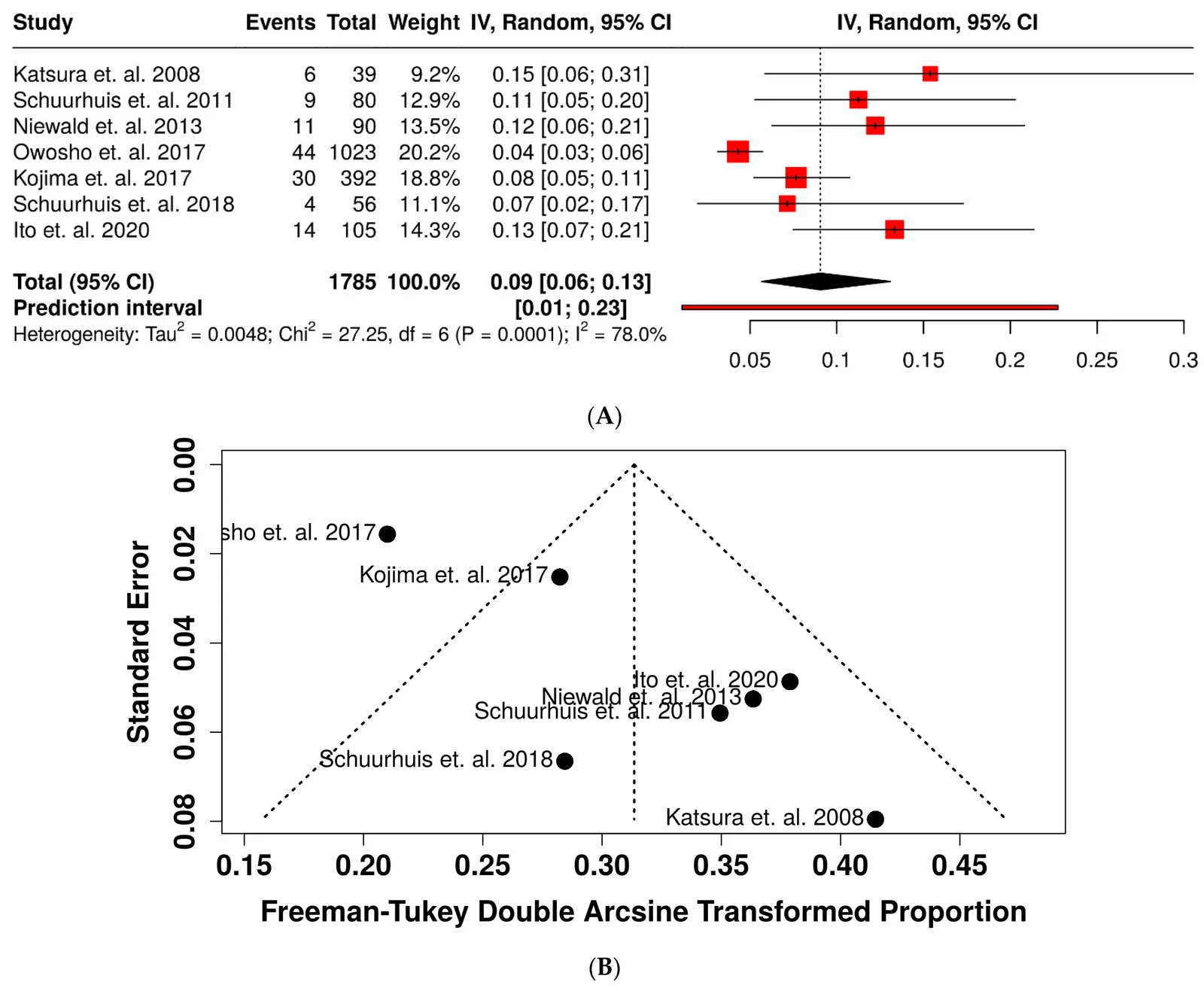
Pooled prevalence of osteoradionecrosis in head and neck cancer patients undergoing radiotherapy. (**A**) Forest plot showing the pooled prevalence of osteoradionecrosis using a random-effects model. Seven studies including 1785 patients and 118 ORN events were included. The pooled prevalence was 9% (95% CI: 6–13%), with substantial heterogeneity across studies (I^2^ = 78.0%; τ^2^ = 0.0048). The prediction interval ranged from 1% to 23%. (**B**) Funnel plot assessing small-study effects in the prevalence meta-analysis. Visual inspection suggested some asymmetry; however, because fewer than ten studies were included and heterogeneity was substantial, publication bias assessment should be interpreted cautiously [24,25,26,28,29,30,32].

Visual inspection of the funnel plot showed some asymmetry, mainly influenced by differences in study size and prevalence estimates. Larger studies, such as Owosho et al. 2017 [28] and Kojima et al. 2017 [29], reported lower prevalence estimates, whereas smaller studies such as Katsura et al. 2008 [24] reported higher prevalence. However, because fewer than ten studies were included and substantial heterogeneity was present, the funnel plot should be interpreted cautiously (Figure 4).

### 3.4. Sensitivity Analysis

**Figure 5:**
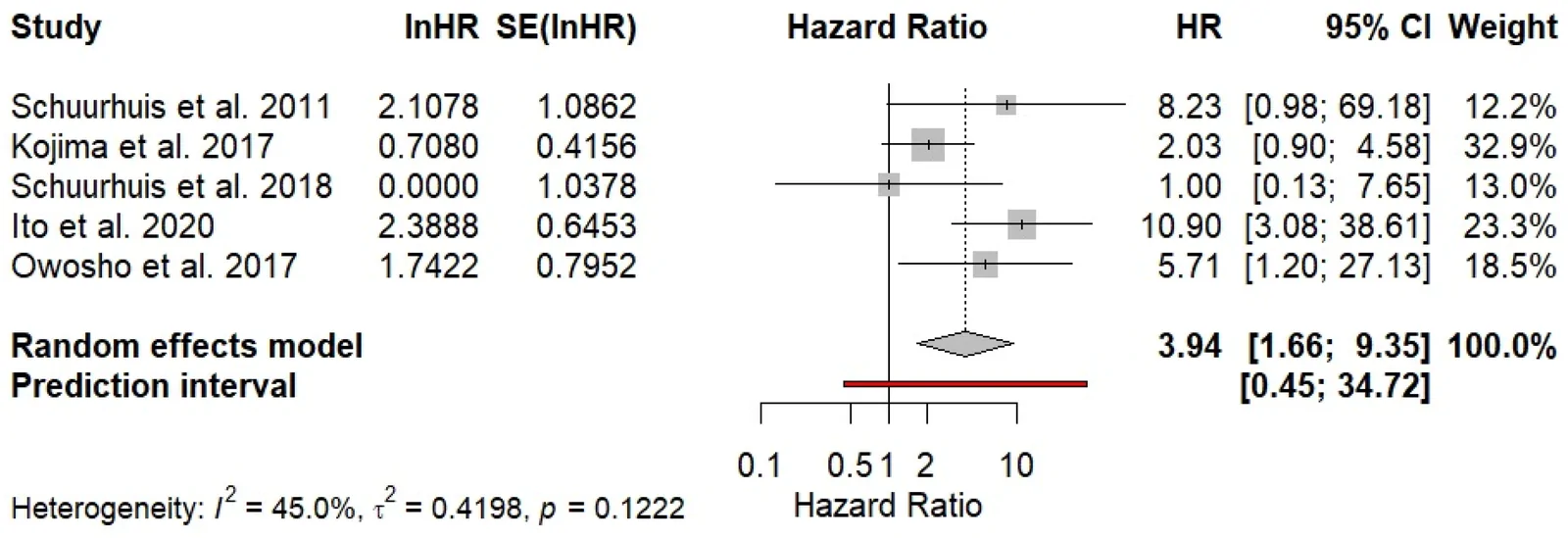
Sensitivity analysis excluding Katsura et al. (2008) [24] for the association between periodontal disease and osteoradionecrosis [25,28,29,30,32].

A sensitivity analysis was performed by excluding Katsura et al. (2008) [24], which represented the study with the largest individual effect estimate in the primary meta-analysis. After removal of this study, the association between periodontal disease and osteoradionecrosis remained statistically significant. The pooled relative effect estimate was 3.94 (95% CI: 1.66–9.35), indicating that periodontal disease or poor periodontal status remained associated with an approximately four-fold higher likelihood of osteoradionecrosis compared with absence of periodontal disease or better periodontal status. Moderate heterogeneity persisted among the remaining studies (I^2^ = 45.0%; τ^2^ = 0.4198; *p* = 0.1222). The prediction interval ranged from 0.45 to 34.72, suggesting that although the pooled association remained significant, the magnitude of the association may vary across future clinical settings. Overall, this sensitivity analysis indicates that the main finding was not driven exclusively by Katsura et al. (2008) [24] (Figure 5).

**Figure 6:**
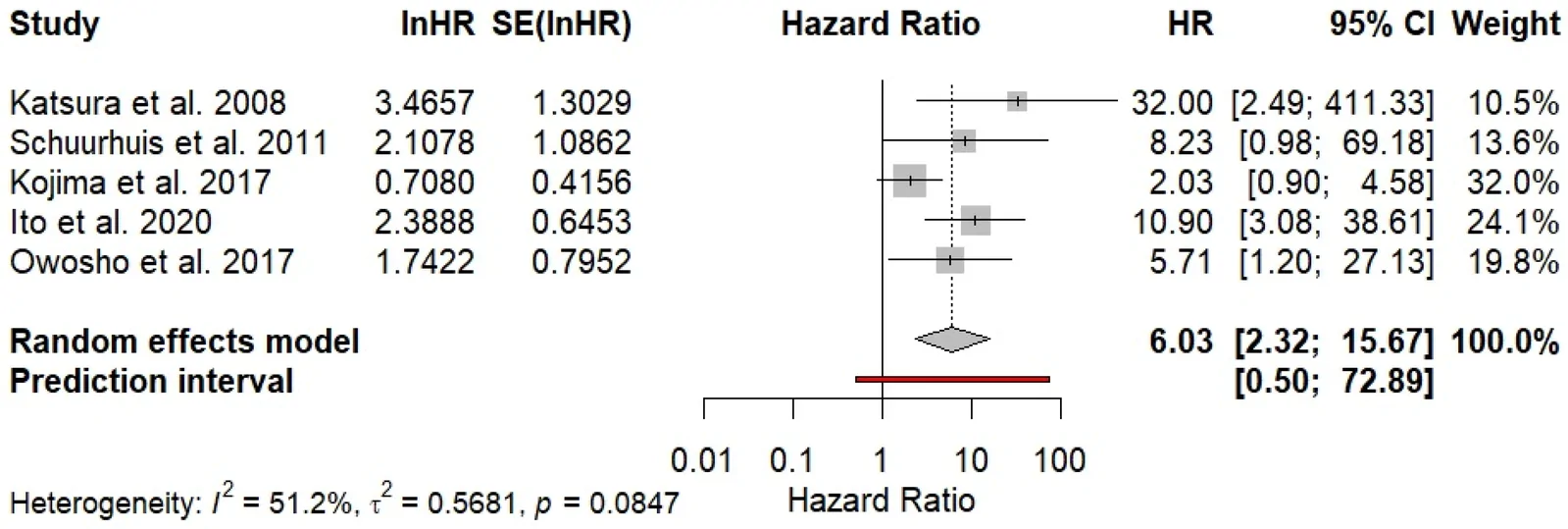
Sensitivity analysis excluding Schuurhuis et al. (2018) [24,25,28,29,30,32].

A second sensitivity analysis was performed by excluding Schuurhuis et al. (2018) [30], the study with the effect estimate closest to the null. After removal of this study, the association between periodontal disease and osteoradionecrosis remained statistically significant. The random-effects model showed a pooled relative effect estimate of 6.03 (95% CI: 2.32–15.67), indicating that periodontal disease or poor periodontal status remained associated with an approximately six-fold higher likelihood of osteoradionecrosis compared with absence of periodontal disease or better periodontal status. Moderate heterogeneity was observed among the remaining studies (I^2^ = 51.2%; τ^2^ = 0.5681; *p* = 0.0847). The prediction interval ranged from 0.50 to 72.89, suggesting that the magnitude of the association may vary across future clinical settings. Overall, the significant association persisted after excluding Schuurhuis et al. (2018) [30], supporting the robustness of the primary meta-analysis (Figure 6).

### 3.5. Hartung–Knapp Sensitivity Analysis

**Figure 7:**
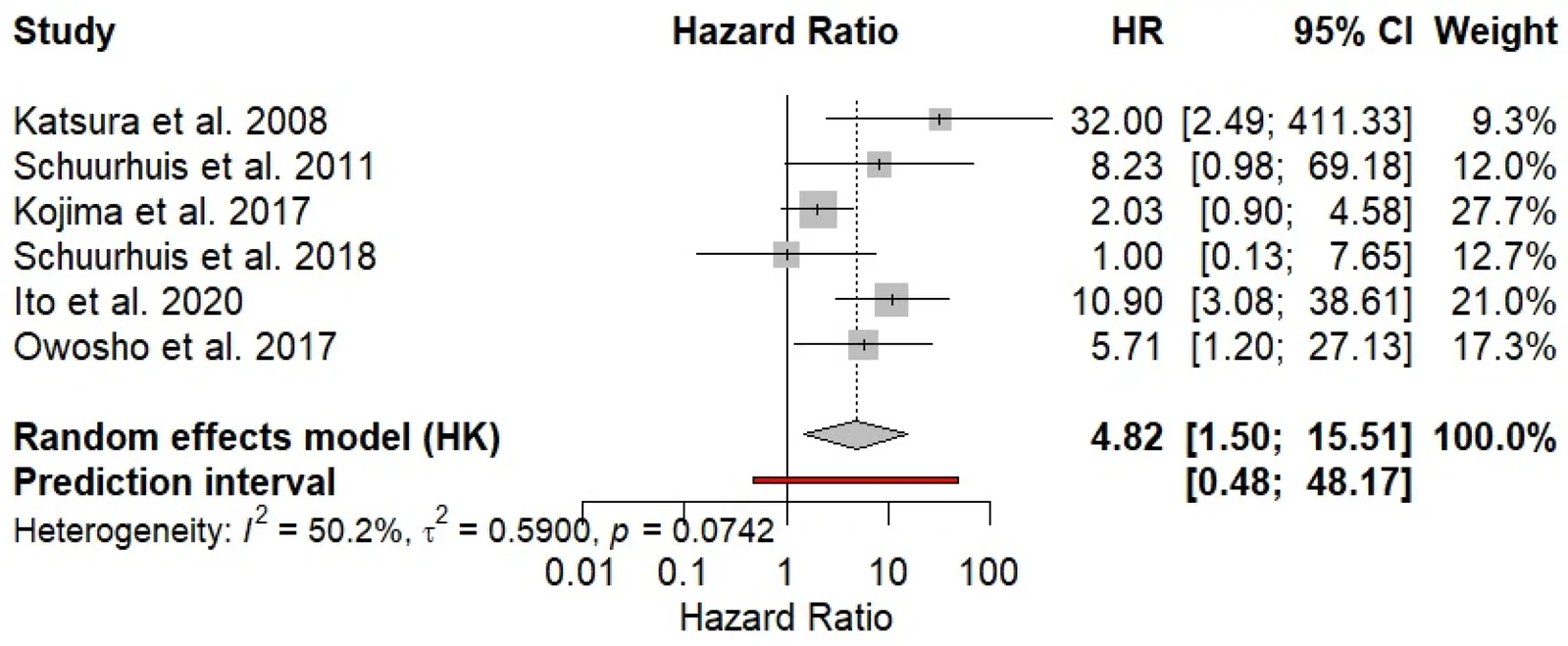
Hartung–Knapp adjusted sensitivity analysis for the association between periodontal disease and osteoradionecrosis [24,25,28,29,30,32].

A Hartung–Knapp adjusted random-effects sensitivity analysis was performed to account for the small number of studies included in the meta-analysis. The association between periodontal disease and osteoradionecrosis remained statistically significant after applying this more conservative approach. The pooled relative effect estimate was 4.82 (95% CI: 1.50–15.51), indicating that periodontal disease or poor periodontal status remained associated with a higher likelihood of osteoradionecrosis compared with absence of periodontal disease or better periodontal status. Moderate heterogeneity was observed across studies (I^2^ = 50.2%; τ^2^ = 0.5900; *p* = 0.0742). The prediction interval ranged from 0.48 to 48.17, suggesting variability in the expected magnitude of the association across future clinical settings. Overall, the Hartung–Knapp analysis supported the robustness of the primary meta-analytic finding (Figure 7).

### 3.6. Leave-One-out Analysis

**Figure 8:**
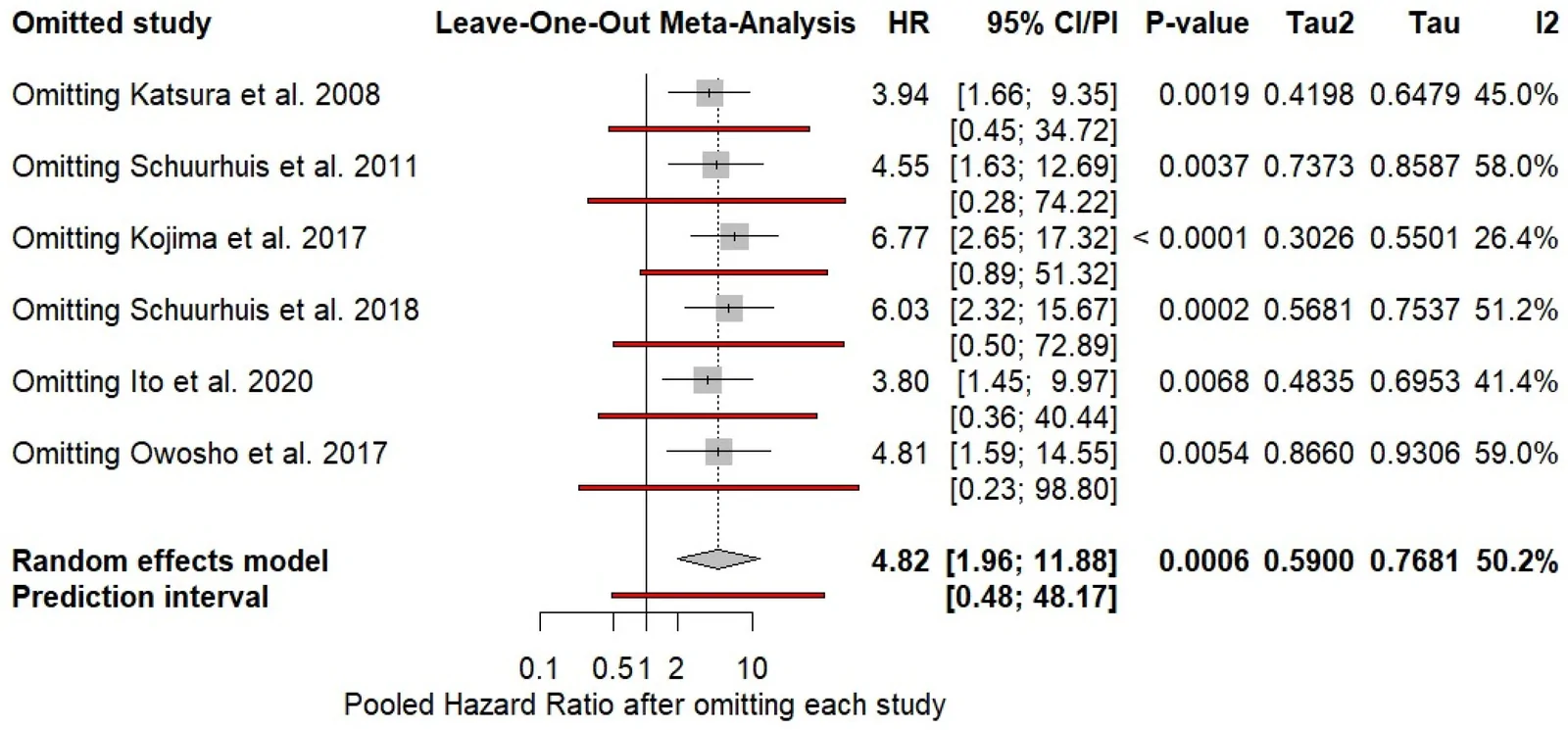
Leave-one-out sensitivity analysis for the association between periodontal disease and osteoradionecrosis [24,25,28,29,30,32].

A leave-one-out sensitivity analysis was conducted by sequentially excluding one study at a time from the primary meta-analysis. The pooled association between periodontal disease and osteoradionecrosis remained statistically significant in all scenarios, with pooled relative effect estimates ranging from 3.80 (95% CI: 1.45–9.97) after excluding Ito et al. (2020) [32] to 6.77 (95% CI: 2.65–17.32) after excluding Kojima et al. (2017) [29]. Between-study heterogeneity ranged from 26.4% to 59.0%. The lowest heterogeneity was observed after omitting Kojima et al. (2017) [29], suggesting that this study may have contributed partially to between-study variability. Overall, the leave-one-out analysis confirmed that the main association between periodontal disease and osteoradionecrosis was robust and was not driven by any single study (Figure 8).

## 4. Discussion

The present meta-analysis provides consistent evidence that periodontal disease is associated with a significantly increased risk of osteoradionecrosis (ORN) in patients with head and neck cancer treated with radiotherapy. Beyond being merely an indicator of poor oral health, our findings support the hypothesis that chronic periodontal inflammation may actively participate in the biological mechanisms that favor the development of ORN. The observed magnitude of effect, with an approximately 4.8-fold higher relative likelihood of ORN among patients with periodontal disease or poorer periodontal status, suggests that this condition may represent a potentially modifiable local factor of high clinical relevance. In addition, the stability of the results after sensitivity analyses, including the Hartung–Knapp adjustment and leave-one-out analysis, reinforces the robustness of the association and reduces the likelihood that it is driven by the disproportionate influence of a single study or by a random finding [24,25].

Although the pooled association was statistically significant, the moderate heterogeneity observed (I^2^ = 50.4%) indicates that the magnitude of the effect may vary across different clinical settings. This variability probably reflects differences in the characteristics of the studied populations, tumor location, radiotherapy modalities and doses, follow-up duration, and the methods used to assess both periodontal disease and ORN. Likewise, factors such as smoking, alcohol consumption, dental extractions, and systemic comorbidities may have contributed to the observed variability. Consequently, periodontal disease should not be understood as an isolated causal factor, but rather as part of a multifactorial process in which radiation-induced injury, chronic infection, and host response interact to determine individual susceptibility to ORN development [28,29].

The included studies provide arguments that strengthen this interpretation. Katsura et al. [24] observed that deterioration of periodontal status after radiotherapy was associated with a higher risk of mandibular ORN, whereas Schuurhuis et al. [25] showed that patients with advanced periodontal disease before treatment had a higher frequency of bone healing problems. Complementarily, Ito et al. [32] demonstrated that stage III periodontitis assessed by computed tomography and increased inflammatory uptake on PET/CT (^18^F-FDG) were associated with a higher probability of developing ORN. Taken together, these findings suggest that not only the structural destruction of periodontal tissues, but also the persistence of an active inflammatory state, may negatively influence the response of irradiated bone to injury and tissue repair [30,32].

The mechanisms currently described in the pathophysiology of osteoradionecrosis provide a biological framework that helps explain the association observed in this meta-analysis. Radiation induces endothelial damage, microvascular alteration, oxidative stress, and progressive fibrosis, resulting in hypoxic, hypovascular, and hypocellular tissue with limited repair capacity. In this context, chronic periodontal inflammation maintains a persistent inflammatory response through the release of proinflammatory cytokines and osteoclastogenic mediators, promotes activation of the RANK/RANKL pathway, and perpetuates a dysbiotic microbiota. The interaction of these processes may further compromise bone remodeling and help explain why patients with periodontal disease show greater susceptibility to developing osteoradionecrosis after radiotherapy [7,36,37].

The pooled ORN prevalence of 9% confirms that this complication remains a clinically important problem, even in the era of intensity-modulated radiotherapy. Nevertheless, the high heterogeneity observed (I^2^ = 78.0%) highlights that its frequency is influenced by multiple factors, including irradiation technique, dose absorbed by the mandible, follow-up duration, and the implementation of preventive dental protocols. In this regard, more recent cohorts treated with IMRT tended to show lower prevalence rates than those reported in earlier studies, probably as a consequence of improvements in radiotherapy planning and the multidisciplinary management of oncology patients, although without completely eliminating the risk of ORN [38,39].

The slight asymmetry observed in the funnel plot should be interpreted with caution because of the limited number of studies available for the meta-analysis, a situation in which this type of analysis has limited ability to detect publication bias. Likewise, the width of the prediction intervals indicates that, although the pooled estimate was significant, the magnitude of the effect may vary considerably in future populations or clinical contexts. Therefore, the results should be interpreted cautiously, especially considering the observational nature of the included studies and the possible influence of residual confounding factors.

From a clinical perspective, these findings reinforce the need to incorporate a comprehensive periodontal evaluation as part of the dental protocol before radiotherapy. Traditionally, pre-radiotherapy management has focused on the elimination of infectious foci and the indication of dental extractions when unavoidable. However, our results suggest that controlling chronic periodontal inflammation may represent a complementary preventive strategy to reduce one of the few potentially modifiable local factors related to the onset of ORN. Consequently, clinical and radiographic assessment of periodontal status, timely periodontal treatment, and long-term periodontal maintenance should be part of the multidisciplinary management of these patients before, during, and after radiotherapy [40,41].

The main strengths of this review include the rigorous application of the PRISMA 2020 recommendations, the systematic search across multiple electronic databases and gray literature sources, and the incorporation of sensitivity analyses to assess the stability of the findings. Nevertheless, several limitations should be considered. First, the available evidence was derived exclusively from observational studies; therefore, residual confounding by factors such as radiation dose, tumor location, smoking, alcohol consumption, systemic conditions, baseline oral health, oncological treatment, and preventive dental protocols cannot be completely excluded.

An additional limitation is the difficulty in isolating the specific contribution of periodontal disease from that of other coexisting oral infectious and inflammatory conditions. Periodontitis is etiologically distinct from dental caries, pulpal or periapical infection, pericoronitis, retained roots, and other odontogenic diseases; however, these conditions frequently coexist in patients with poor oral hygiene, xerostomia, reduced salivary protection, limited self-care capacity, and a high overall oral disease burden. In some included studies, periodontal disease was evaluated within broader categories of oral infectious foci or general dental status that also included carious lesions, periapical disease, and teeth requiring extraction. Consequently, the observed association may partly reflect the combined influence of periodontal inflammation, other odontogenic infections, and compromised oral health rather than an effect attributable exclusively to periodontitis.

Dental extraction represents a particularly complex factor because it may be performed as a consequence of advanced periodontitis, extensive caries, periapical infection, or other dental diseases, while also acting as a potential precipitating event for ORN, particularly when performed after radiotherapy. Depending on its indication and timing, extraction may therefore function as a confounding factor, an intermediate event, or a marker of underlying oral disease severity. Because the presence of coexisting dental diseases and the indication and timing of dental extractions were not consistently reported or adjusted for across the included studies, residual confounding and overlap between periodontal and non-periodontal oral exposures cannot be excluded.

Second, and particularly relevant to the interpretation of the present findings, periodontal exposure was not defined uniformly across the included studies. Some studies assessed periodontal status using clinical parameters, including probing depth, clinical attachment loss, plaque scores, periodontal pocket progression, or periodontal inflamed surface area. Other studies relied on radiographic alveolar bone loss, panoramic radiographic scores, computed tomography-based periodontal staging, or PET/CT inflammatory uptake, whereas periodontal disease was sometimes included within broader categories of oral infectious foci or general dental status. These approaches represent related but not diagnostically interchangeable constructs according to the contemporary periodontal classification. Radiographic bone loss primarily reflects cumulative periodontal destruction and does not necessarily indicate current inflammatory activity, whereas broader oral-foci definitions may also include carious, endodontic, and other non-periodontal conditions.

Because most studies did not provide the complete clinical and radiographic information required to apply a uniform contemporary case definition, retrospective classification according to periodontitis stage, grade, and extent was not possible. This variability may have introduced exposure misclassification, contributed to the observed methodological heterogeneity, and affected the magnitude and precision of the pooled association. Additional heterogeneity in ORN definitions, radiotherapy dosimetry, timing of periodontal assessment, oral hygiene, systemic status, and preventive protocols also limited the feasibility of subgroup analyses and meta-regression.

The risk-of-bias findings should be interpreted at the domain level rather than solely according to study design. Although several studies were retrospective, retrospective data collection does not necessarily introduce substantial bias in every methodological domain. Most studies used defined clinical cohorts, documented radiotherapy histories, and clinical, radiographic, or imaging records to assess periodontal status and ORN. These characteristics supported low-risk judgments in domains such as participant selection, missing data, outcome measurement, and selection of the reported result. Nevertheless, the principal methodological concern remained residual confounding because radiation dose, tumor location, dental extractions, smoking, alcohol consumption, baseline oral health, coexisting odontogenic infections, and oncological treatment were not uniformly incorporated into adjusted analyses. Variability in the definition and timing of periodontal exposure also introduced concern regarding exposure classification. Therefore, the risk-of-bias assessment should not be interpreted as demonstrating the absence of bias or a high certainty of evidence, but rather as indicating that no major methodological flaw was identified in most evaluated domains.

In conclusion, the available evidence suggests an association between broadly defined periodontal disease or poor periodontal status and a greater likelihood of osteoradionecrosis in patients undergoing radiotherapy for head and neck cancer. However, the observational nature of the included studies, the non-uniform assessment of periodontal exposure, the coexistence of other oral infections, and the potential influence of residual confounding preclude the establishment of a definitive causal relationship. The pooled estimate cannot determine whether the observed association is attributable specifically to periodontitis or partly to the broader burden of oral infection, dental disease, and related interventions. Therefore, the findings should be interpreted as an association with broadly defined periodontal disease or compromised oral health rather than as an effect specific to a standardized diagnosis of periodontitis or to a particular periodontitis stage or grade.

Nevertheless, the findings provide a clinical rationale for considering comprehensive periodontal assessment, preventive dental care, and long-term oral surveillance within the multidisciplinary management of these patients. Future prospective studies should apply contemporary and standardized periodontal case definitions, including periodontitis stage, grade, and extent, and should separately record dental caries, pulpal and periapical infections, pericoronitis, retained roots, oral hygiene indicators, and dental extractions, including their clinical indication and timing in relation to radiotherapy. Consistent ORN diagnostic criteria, detailed dosimetric characterization, adequate control of confounding factors, and longitudinal follow-up will also be necessary to determine whether optimization of periodontal and general oral health can meaningfully reduce the incidence of this severe complication.

## 5. Conclusions

This systematic review and meta-analysis found that periodontal disease or poor periodontal status was associated with a greater likelihood of osteoradionecrosis in patients with head and neck cancer undergoing radiotherapy. However, the observational nature of the included studies, moderate heterogeneity, variability in periodontal definitions and assessment timing, and the possibility of residual confounding prevent causal conclusions. The findings provide a rationale for considering comprehensive periodontal assessment and long-term oral surveillance within multidisciplinary care, although it remains uncertain whether periodontal treatment directly reduces ORN incidence. Prospective studies using standardized periodontal and ORN diagnostic criteria and adequate adjustment for relevant confounding factors are needed to confirm the association and determine its clinical and preventive implications.

## Figures and Tables

**Table 1:** Characteristics of the studies included in the systematic review and meta-analysis.

Author/Year	Study Design	Population and Cancer Type	Radiotherapy/Oncological Treatment	Timing of Periodontal Assessment/Follow-Up	Periodontal or Oral Health Assessment	Main Outcome Evaluated	ORN Events/Prevalence	Main Qualitative Finding
Katsura et al., 2008 [24]	Retrospective longitudinal study	39 patients with head and neck cancer whose radiation fields included the teeth and mandible	External-beam radiotherapy; mandibular/oral cavity radiation exposure assessed clinically	≥3 years of follow-up after radiotherapy; ORN onset occurred 18–51 months after RT	Periodontal pocket depth, dental plaque score, alveolar bone loss level, and radiographic periodontal status. Advanced periodontal deterioration included periodontal pocket depth ≥ 5 mm, plaque score ≥ 40%, alveolar bone loss ≥ 60%, and radiographic periodontal status grade 3	Mandibular osteoradionecrosis	6/39 patients developed ORN	Oral health status before RT was not clearly associated with ORN, whereas poor oral/periodontal status after RT, particularly advanced radiographic periodontal status, was strongly associated with subsequent ORN. This study highlights the importance of continuous periodontal maintenance after radiotherapy.
Schuurhuis et al., 2011 [25]	Retrospective study	80 dentate or partially dentate patients with head and neck cancer scheduled for curative radiotherapy	Curative head and neck radiotherapy after pre-radiation dental screening and dental treatment when indicated	ORN occurred on average 13.5 months after RT; range 3–31 months	Pre-radiation dental screening for oral foci, especially periodontal pockets ≥ 6 mm. Oral foci included severe periodontitis, caries, and teeth requiring extraction	Osteoradionecrosis	9/80 patients developed ORN	Oral foci were common before RT, with periodontal disease being the predominant focus. Eight of nine ORN cases had severe periodontal disease at dental screening. Patients with periodontal pockets ≥ 6 mm showed increased ORN risk, particularly when periodontally compromised teeth were initially preserved rather than extracted.
Niewald et al., 2013 [26]	Retrospective study	90 patients with oral neoplasms treated with radiotherapy	Conventional radiotherapy; total dose range reported between 36 and 76.8 Gy	Retrospective post-RT evaluation	Dental status, oral hygiene, biofilm, dental calculus, chronic periodontitis, mild/moderate attachment loss, and severe attachment loss	Infected osteoradionecrosis of the lower jaw	11/90 patients developed infected ORN	Chronic periodontitis and poor dental/oral hygiene were frequent in the cohort. However, periodontal variables such as chronic periodontitis with mild/moderate or severe attachment loss were not reported with extractable comparative effect estimates for ORN. Therefore, the study contributes prevalence and qualitative evidence but cannot be used in the main association meta-analysis.
Khaw et al., 2014 [27]	Prospective pilot study	33 patients with head and neck cancer undergoing radiotherapy; 28 evaluable for periodontitis	Head and neck radiotherapy	Periodontitis assessed in relation to mucositis during RT	Clinical and radiographic periodontitis assessment, including alveolar bone loss, periodontal pocket depth, clinical attachment level, and inflammatory biomarkers in gingival crevicular fluid	Radiation-induced oral mucositis	Not applicable; ORN was not evaluated	The study explored whether periodontitis was associated with oral mucositis severity. No statistically significant association was demonstrated, although patients with mucositis showed trends toward greater bone loss, probing depth, and clinical attachment loss. This study supports the biological plausibility of periodontal inflammation contributing to RT-related oral toxicity but does not provide ORN data.
Owosho et al., 2017 [28]	Retrospective cohort with matched case–control analysis	1023 patients with oral cavity or oropharyngeal cancer treated with IMRT	Intensity-modulated radiation therapy; oral cavity and oropharyngeal cancer patients treated from 2004 to 2013	Median follow-up 52.5 months	Pre-RT periodontal status assessed on panoramic radiographs and scored from 0 to 3 according to periodontal bone loss: 0 = no bone loss; 1 = mild; 2 = moderate; 3 = severe	Osteoradionecrosis of the jaw	44/1023 patients developed ORN; prevalence 4.3%	Poor periodontal status was significantly associated with ORN in univariate matched analysis. Most ORN cases occurred spontaneously, without preceding dental trauma. In multivariate analysis, alcohol use and radiation dose remained significant, indicating that periodontal disease may interact with radiation and behavioral risk factors.
Kojima et al., 2017 [29]	Multicenter retrospective observational study	392 patients with head and neck cancer who underwent radiotherapy	Radiotherapy > 50 Gy; oral/oropharyngeal cancers and other head and neck sites included	ORN cumulative occurrence assessed by Kaplan–Meier and Cox regression	Dental status before and after RT, including severe marginal periodontitis before RT, periapical periodontitis, dental caries, carious stump, and post-RT tooth extraction	Osteoradionecrosis of the jaws	30/392 patients developed ORN	Severe marginal periodontitis before RT showed a trend toward increased ORN risk, although the confidence interval crossed the null. The original study identified oral/oropharyngeal cancer, periapical periodontitis, and post-RT tooth extraction as independent risk factors. The study emphasizes that both baseline dental infection and post-RT dental deterioration may contribute to ORN development.
Schuurhuis et al., 2018 [30]	Prospective 2-year follow-up study	56 dentate or partially dentate patients with oral or oropharyngeal cancer	IMRT or chemoradiotherapy with pre-radiation dental screening and treatment of oral foci	2-year follow-up after IMRT/CHIMRT	Periodontal pockets ≥ 6 mm, periodontal inflamed surface area (PISA), periodontal pocket progression, and oral/dental morbidity after RT	Osteoradionecrosis and bone healing problems	4/56 patients developed ORN	For strict ORN, the association with periodontal pockets ≥ 6 mm was not evident. However, higher PISA and more severe periodontal inflammation before IMRT/CHIMRT were associated with bone healing problems. This study suggests that periodontal inflammatory burden may be clinically relevant even when ORN alone is uncommon.
Morais et al., 2020 [31]	Prospective observational cohort	61 patients with head and neck squamous cell carcinoma receiving RT or radiochemotherapy	RT or RT combined with chemotherapy; preventive oral care program plus daily photobiomodulation therapy	During RT, with serial assessments across RT sessions	Preventive oral care protocol including plaque control, oral hygiene monitoring, removal of infection foci, restoration of carious lesions, periodontal therapy, fluorotherapy, oral hydration, denture removal at night, and PBMT	Oral mucositis, pain, dysphagia, odynophagia, dysgeusia, xerostomia, quality of life, and RT interruptions	ORN not evaluated as main outcome	The preventive oral care program improved oral health parameters, including plaque control and gingival inflammation, and was associated with satisfactory control of oral adverse effects. Interruption of RT due to oral mucositis occurred in only a small proportion of patients. The study is useful for discussing preventive strategies but not for estimating periodontal disease–ORN association.
Ito et al., 2020 [32]	Retrospective study	105 patients with oral cavity or oropharyngeal squamous cell carcinoma treated with RT	Radiotherapy/IMRT; all patients underwent pretreatment ^18^F-FDG PET/CT	Post-treatment diagnosis of ORN after RT; pretreatment imaging used for prediction	Periodontitis staged on CT using the 2018 periodontitis staging framework; PET/CT periodontal inflammatory uptake measured by SUVmax	Osteoradionecrosis	14/105 patients developed ORN; prevalence 13.3%	Stage III periodontitis on CT was strongly associated with ORN. SUVmax > 2.1 also predicted ORN, suggesting that active periodontal inflammation identified by PET/CT may have prognostic value. For the primary meta-analysis, CT-based periodontitis stage III was selected because it directly represents periodontal disease severity.
Benito-Ramal et al., 2024 [33]	Retrospective observational pilot cohort	175 patients with head and neck cancer treated with surgery, RT, chemotherapy, immunotherapy, or combined modalities	Mixed oncological therapies, including RT-containing regimens and non-RT therapies	Retrospective chronic complication assessment	Dental disease and periodontal disease recorded as chronic oral complications; oral hygiene and prior/posterior invasive dental treatment also considered	Chronic oral complications, dental disease, periodontal disease, xerostomia, mucositis, infections, ORN/OQN, and other sequelae	ORN/OQN reported together: 13/175; 7.43%	Chronic oral complications were highly prevalent. Dental disease, periodontal disease, and xerostomia were among the most frequent chronic complications. However, because periodontal disease was treated as an outcome and ORN was combined with osteochemonecrosis, the study is not suitable for the main association meta-analysis or ORN-only prevalence meta-analysis.

Abreviaturas: HNC, head and neck cancer; RT, radiotherapy; ORN, osteoradionecrosis; CAL, clinical attachment loss; BOP, bleeding on probing; PISA, periodontal inflamed surface area; PBMT, photobiomodulation therapy; SCC, squamous cell carcinoma; OQN, osteochemonecrosis.

## Data Availability

The raw data supporting the conclusions of this article will be made available by the authors, without undue reservation.

## Supplementary Materials

The following supporting information can be downloaded at https://www.mdpi.com/article/10.3390/dj14080520/s1, Table S1: PRISMA 2020 Checklist.
